# How to Complete a Gynecological Robot-Assisted Retroperitoneal Lymph Node Dissection with a High-Quality Experience: 180 Cases in a Single Center

**DOI:** 10.3390/jcm15197746

**Published:** 2026-10-08

**Authors:** Zixi Liu, Lei Dou

**Affiliations:** Department of Gynecology, The First Hospital of China Medical University, Shenyang 110002, China; zixi991227@163.com

**Keywords:** Da Vinci robot surgical system, pelvic lymphadenectomy, para-aortic lymphadenectomy, retroperitoneal lymphadenectomy

## Abstract

**Background**: This study seeks to provide a comprehensive overview of robotic retroperitoneal lymphadenectomy in patients with endometrial cancer, including surgical techniques, quality enhancement, and complication reduction. **Methods**: A cohort of 180 endometrial cancer patients who underwent retroperitoneal lymphadenectomy with the Da Vinci robotic surgical system in the gynecology department from November 2017 to June 2023 was compared to 195 patients who underwent laparoscopic retroperitoneal lymphadenectomy during the same period as controls. **Results**: In surgical interventions, the robotic retroperitoneal lymphadenectomy demonstrated superior results compared to laparoscopic surgery, including shorter surgical duration (165 vs. 180 min, *p* = 0.03), reduced intraoperative bleeding 45 vs. 65 mL, *p* = 0.04, lower incidence of postoperative complications 19 vs. 33, *p* = 0.03, and higher rates of complete lymph node resections 25 vs. 20, *p* = 0.02. Notably, the robotic approach excelled in the retrieval of high para-aortic lymph nodes, particularly in the superior region of the inferior mesenteric artery 5 vs. 3, *p* = 0.04. In postoperative recovery, the robot group also exhibited significant advantages over the laparoscopic group in various outcomes, including postoperative anal exhaust time 47 vs. 55, *p* = 0.02, first time out of bed activity time 8 vs. 9, *p* = 0.04, postoperative hospital stay 2 vs. 4, *p* = 0.01, and VAS score 4.0 vs. 4.6, *p* = 0.04, 2.9 vs. 3.4, *p* = 0.03, 1.1 vs. 1.8, *p* = 0.04. In surgical outcomes, there was no statistically significant variance observed between the two groups. **Conclusions**: Robot retroperitoneal lymphadenectomy still follows the classic surgical steps of oncology principles. Summarizing surgical techniques can effectively improve surgical quality and reduce surgical complications.

## 1. Introduction

Endometrial cancer, ranking as the sixth most prevalent malignant neoplasm among women, carries a lifetime risk of approximately 3%. In the year 2020, the global incidence of this disease reached 417,000 new cases, predominantly affecting individuals with a median age of 61 years at diagnosis [1]. Notably, advanced age and obesity have been identified as two prominent risk factors associated with the development of endometrial cancer [2,3]. The therapeutic modalities employed for managing this condition encompass surgical intervention, radiotherapy, chemotherapy, endocrine therapy, among others, with surgical intervention serving as the primary treatment modality [4]. Lymph node metastasis is a prevalent and significant pathway for the spread of endometrial cancer, occurring either through a stepwise sequential progression from pelvic to para-aortic lymph nodes or through a skip-like manner directly to para-aortic lymph nodes without involvement of pelvic lymph nodes. Consequently, in the context of endometrial cancer, the comprehensive examination of retroperitoneal lymph nodes (including both pelvic and para-aortic lymph nodes) to ascertain the presence of tumor metastasis holds immense importance in establishing accurate surgical pathological staging and informing postoperative adjuvant therapy [5,6].

Classic retroperitoneal lymph node dissection is frequently conducted via open surgery, involving a substantial incision, resulting in elevated complications and incomplete resection, thereby impacting the patient’s prognosis to a certain extent [7]. The increasing prevalence of minimally invasive laparoscopic surgery has led to the gradual recognition of its safety and effectiveness in retroperitoneal lymph node dissection [8,9]. The advent of the Da Vinci robotic surgical system has introduced a novel era of precise minimally invasive surgery. Since its approval by the US Food and Drug Administration (FDA) in 2005 for gynecological surgery, the Da Vinci robotic surgical system has garnered significant attention and utilization on a global scale [10,11]. In comparison to open and laparoscopic procedures for retroperitoneal lymph node dissection, this robotic system offers notable benefits, including reduced surgical duration, decreased intraoperative bleeding, diminished complications, and expedited postoperative recuperation [12]. Consequently, these advantages further facilitate the adoption of the Da Vinci robotic surgical system in endometrial cancer surgery. Hence, the objective of this study is to conduct a comparative analysis of the safety and efficacy of the Da Vinci robotic surgical system and laparoscopy in the surgical management of endometrial cancer. Additionally, this research aims to organize surgical particulars, consolidate surgical techniques, and enhance overall surgical outcomes.

## 2. Materials and Methods

### 2.1. Research Design and Population

This research constitutes a retrospective clinical study conducted at a solitary medical center. Following a thorough examination and endorsement by the Ethics Committee of the First Affiliated Hospital of China Medical University. All participants encompassed within this investigation were duly apprised of pertinent information and provided informed consent. A total of 180 patients diagnosed with endometrial cancer were chosen for the study, specifically those who had undergone retroperitoneal lymph node dissection utilizing the Da Vinci robotic surgical system (hereinafter, it is referred to as the robot) within the gynecology department between November 2017 and June 2023. Additionally, 195 endometrial cancer patients who had undergone laparoscopic retroperitoneal lymph node dissection during the same study period were selected as controls. All surgical procedures for endometrial cancer are conducted exclusively by a unified cohort of physicians who possess formal training, requisite qualifications, and exceptional proficiency in executing robotic, laparoscopic, or open surgery techniques. The inclusion criteria encompass the following: ① all patients must have received a pathological diagnosis; ② retroperitoneal lymph node dissection is mandatory in accordance with the NCCN guidelines; ③ willingness to undergo either robot-assisted or laparoscopic surgery. The exclusion criteria encompass the following: ① patients diagnosed with clinical stage III/IV endometrial cancer exhibiting lesions extending beyond the uterus; ② individuals with severe heart, liver, kidney, or coagulation dysfunction, as well as those suffering from severe pelvic inflammatory disease; ③ participants who have undergone radiotherapy or chemotherapy or have opted for exclusive radiotherapy or chemotherapy.

Assignment to the robotic or laparoscopic group was not predetermined but was based on Patient’s willingness. All patients who meet the surgical indications undergo a standardized informed consent procedure before surgery, with the attending physician providing a detailed introduction to the advantages and disadvantages, cost differences, and expected benefits of robotic surgery and laparoscopic surgery. Patients make choices based on their own economic situation and personal preferences. All enrolled patients underwent surgery by the same group of gynecologists, with a learning curve inflection point of 20 cases, and achieved a relatively stable level of operation after 20 surgeries. In addition, among the enrolled patients, those with intraoperative suspicion of metastatic lymph nodes (enlarged, firm, or fixed nodes) or postoperative pathologically confirmed positive nodes were not excluded, provided they met the inclusion criteria.

### 2.2. Surgical Methods

The Da Vinci Surgical Si™ system by Intuitive Surgical, located in Sunnyville, CA, USA, is utilized by the robot team. The laparoscopic group employs a high-definition three-dimensional laparoscopic system (Karl Storz, Thuringen, Germany) as their surgical equipment. The surgical procedure adheres strictly to the NCCN Clinical Practice Guidelines for Uterine Tumors [13]. The surgical approach encompasses total hysterectomy outside the fascia, double appendectomy, pelvic and para-aortic lymph node dissection, and greater omentum biopsy, which are determined based on preoperative clinical examination, imaging assessment, and a comprehensive evaluation of the surgical context. In cases of low-grade endometrioid adenocarcinoma, where myometrial invasion is less than half, absence of family history of breast cancer/ovarian cancer or Lynch syndrome, and absence of ovarian involvement and extrauterine metastasis as determined through preoperative examination and intraoperative evaluation, the option of ovarian preservation may be considered, while bilateral fallopian tubes may be removed. However, patients with pathological results indicating endometrial serous carcinoma, clear cell carcinoma, carcinosarcoma, and undifferentiated carcinoma should undergo simultaneous omental biopsy.

### 2.3. Lymph Node Assessment Strategy

During the study period (2017–2023), systematic pelvic and para-aortic lymphadenectomy was the standard lymph node assessment method in our institution for all endometrial cancer patients undergoing minimally invasive surgery. The primary reason was technical: the Da Vinci Si™ robotic system (Intuitive Surgical, Sunnyvale, CA, USA) used throughout this period does not possess near-infrared fluorescence imaging capability (Firefly™ technology), which is essential for intraoperative indocyanine green (ICG)-based sentinel lymph node (SLN) mapping. Our laparoscopic equipment similarly lacked fluorescence imaging modules. Therefore, SLN biopsy was not feasible in either group during the study period. Consequently, all enrolled patients underwent systematic pelvic and para-aortic lymphadenectomy, which also aligned with NCCN guideline recommendations for high-risk histologies. This uniform approach ensured consistent surgical staging between the robotic and laparoscopic groups, eliminating potential confounding from mixed nodal assessment strategies.

### 2.4. Patient Baseline Characteristics and Evaluation Indicators

All variables were defined prior to data extraction based on clinical relevance and literature review. Baseline characteristics include age, BMI, FIGO staging, pathological type, comorbidities, ECOG score, and history of abdominal surgery. These were collected for descriptive comparison only and were not used as adjustment variables unless they showed imbalance (*p* < 0.05) between groups.

The study assessed various parameters. Primary outcomes include surgical time (specifically docking time and lymph node dissection time), the number of excised lymph nodes (specifically pelvic lymph nodes and para-aortic lymph nodes), and intraoperative blood loss. Secondary outcomes include anal exhaust time, first time out of bed activity, postoperative hospital stay, VAS score at 12 h, 24 h, and 72 h, surgical quality evaluation indicators such as surgical complications, total survival, disease-free survival, and the need for postoperative supplementary radiotherapy and chemotherapy, as well as the total cost. Considering that some patients with preoperative assessment of stage I or II may observe suspicious lymph node positivity during surgery or have lymph node positivity detected by postoperative pathology, we also included these patients in the analysis and marked them in the results.

### 2.5. Statistical Processing

Statistical analysis was conducted using the SPSS 24.0 version (IBM Inc., Armonk, NY, USA). The analysis revealed that a *p*-value < 0.05 is deemed statistically significant. Continuous variables were assessed using the student *t*-test, while categorical variables were evaluated using the *t*-test χ^2^ Inspection. Non-parametric statistics were performed using the Mann–Whitney U test and Fisher’s exact test.

### 2.6. Follow-Up

Out of the total sample size of 375 patients, 5 individuals succumbed to causes unrelated to the study (specifically, 2 due to traffic accidents, 1 due to cerebral hemorrhage, 1 due to coronary heart disease, and 1 due to pneumonia), while 11 patients were lost to follow-up. Consequently, postoperative follow-up data was obtained for a total of 359 patients. Among these, 174 patients belonged to the robot group, whereas 185 patients belonged to the laparoscopic group, resulting in follow-up rates of 96% and 94.9% respectively. The overall median follow-up duration was 38 months, ranging from 5.0 to 67.0 months. The patient will undergo regular follow-up appointments every three months for a duration of two years post-surgery. These appointments will primarily involve assessing symptoms, conducting systemic and gynecological pelvic examinations, monitoring tumor markers, performing gynecological ultrasound, and conducting additional imaging examinations as necessary based on the patient’s condition [14,15]. The follow-up process will also encompass gathering information regarding the patient’s survival status, recurrence status, and other relevant factors. Two gynecologists, who have received appropriate training, will review and input the same case data, scrutinize any suspicious parameters, and subsequently enter them into a standardized research database.

## 3. Methods for Achieving Precise and Efficient Retroperitoneal Lymph Node Dissection in Robotic Surgery

**Figure 1:**
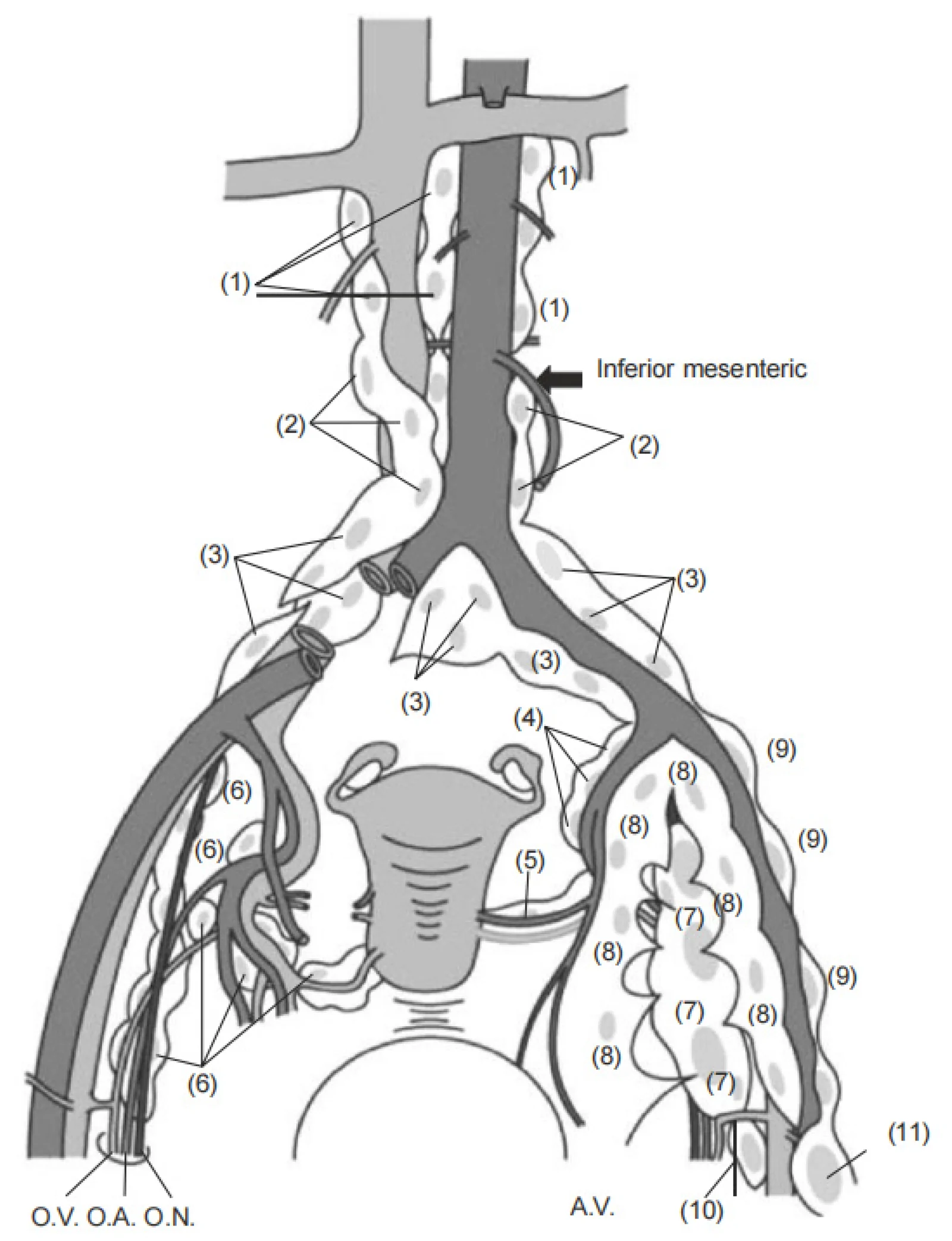
Grouping and naming of retroperitoneal lymph nodes. Periaortic lymph nodes: (1) the superior region of the inferior mesenteric artery, (2) the inferior region of the inferior mesenteric artery; pelvic lymph nodes: (3) common iliac lymph nodes, (4) anterior sacral nodules, (5) para-uterine artery lymph nodes, (6) main ligament lymph nodes, (7) obturator lymph nodes, (8) iliac internal lymph nodes, (9) external iliac lymph nodes, (10) distal iliac circumflex lymph nodes, (11) external iliac circumflex lymph nodes. AV unnamed vein, OA obturator artery, ON obturator nerve, OV obturator vein. The EIN is situated laterally to the external iliac artery, below the branch of the common iliac artery. The CINDEINs represent the most distal iliac lymph nodes. The IINs are positioned medially to the internal iliac artery, below the bifurcation of the common iliac artery, amidst the iliac vessels. The ONs are located superior to the obturator nerve and inferior to the iliac vessels. The CINDONs are the furthest obturator lymph nodes, situated below the unnamed vein. The PUNs are associated with either uterine arteries or veins. The deep obturator lymph nodes, also known as CLNs, are situated beneath the obturator nerve. The medial side of the internal iliac artery, below the bifurcation of the common iliac artery, is the location of the SNs. Positioned between the bifurcation of the common iliac artery and the bifurcation of the aorta are the CINs.

**Figure 2:**
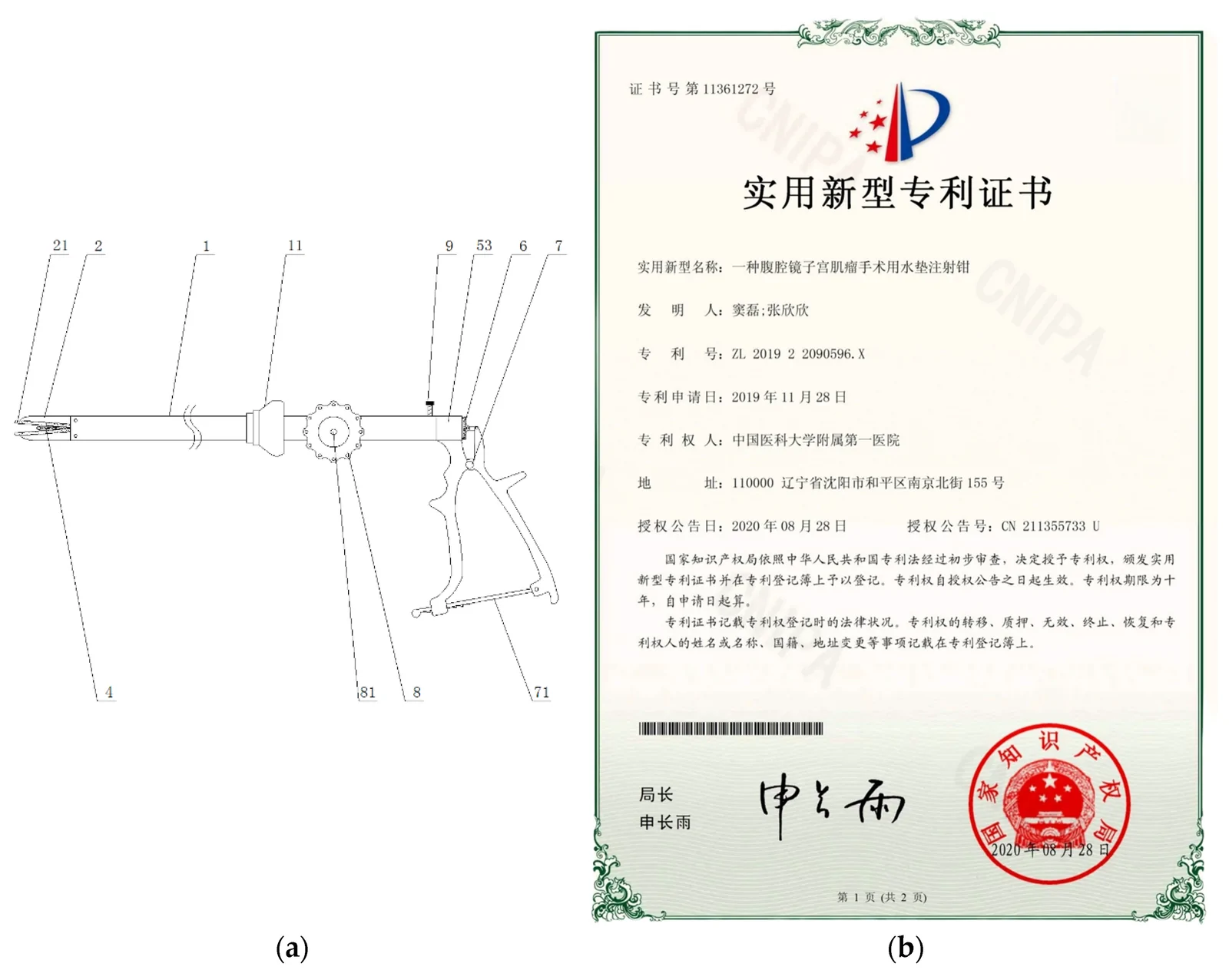
(**a**) Water cushion injection forceps and (**b**) Chinese utility model patent certificate for the water cushion injection forceps. Patent No. ZL 2019 2 2090596.X; Inventor: Lei Dou; Patentee: The First Hospital of China Medical University; Date of Authorization: 28 July 2020. The certificate is issued by the National Intellectual Property Administration of China. The patented device is designed for hydro-dissection of fixed metastatic lymph nodes during robotic surgery.

While the fundamental steps of pelvic and para-aortic lymphadenectomy follow established oncological principles, our approach incorporates several refinements that distinguish it from conventional descriptions. Our 180-case experience presents a step-by-step technical solution that integrates: a refined 9-zone anatomical zoning system (Figure 1), a “water-cushion” patent technique for challenging dissections (Figure 2), zone-specific instrument strategies, and an “easy-first” operational philosophy. These technical details, refined from 180 consecutive cases, aim to provide a reproducible quality improvement framework for surgeons adopting robotic lymphadenectomy.

### 3.1. The Categorization and Nomenclature of Retroperitoneal Lymph Nodes

The surgical procedure known as retroperitoneal lymph node dissection for endometrial cancer encompasses the dissection of both pelvic and para-aortic lymph nodes. The pelvic lymph nodes can be further categorized into nine distinct regions, namely the distal iliac lymph nodes (CINDEINs), external iliac lymph nodes (EINs), iliac circumflex to distal obturator lymph nodes (CINDONs), iliac vascular lymph nodes (IINs), obturator lymph nodes (ONs), and para-uterine artery lymph nodes (PUNs); the main ligament lymph nodes (CLNs), sacral anterior lymph nodes (SNs), and common iliac lymph nodes (CINs) are anatomical structures of interest. The para-aortic lymph nodes, situated between the bifurcation level of the aorta and the renal vein level, also warrant attention. These lymph nodes can be categorized into three groups: the anterior group of the abdominal aorta, the posterior group of the abdominal aorta, and the lateral group of the left and right abdominal aorta. Additionally, they can be further divided into two regions: the superior area of the inferior mesenteric artery and the inferior area of the inferior mesenteric artery (Figure 1).

### 3.2. Preoperative Comprehensive Assessment of Retroperitoneal Lymph Nodes

Integrating diverse imaging modalities including CT, MRI, PET/CT, etc., can offer an initial appraisal of lymph nodes and facilitate the formulation of a surgical strategy.

### 3.3. The Process of Selecting the Appropriate Body Position and Puncture Hole Position

During the administration of general anesthesia, the patient is positioned in the Trendelenburg position, with the arms effectively immobilized in close proximity to the body. A meticulous application and securement of the surgical sheet is undertaken to optimize the surgical area, limit patient movement, and minimize the risk of nerve damage. The legs are positioned at an angle ranging from 60 to 90 degrees, which allows for the convenient placement of the surgical cart between them. This positioning brings the cart closer to the abdominal surgical site and ensures that there is no accidental contact with the robotic arm when the legs are elevated into the stone cutting position. Throughout the surgical procedure, the intra-abdominal pressure of carbon dioxide gas was increased to approximately 12–14 mmHg. To prevent the dissemination of tumor cells resulting from uterine cup elevation, the uterus was manipulated by suturing both sides of the uterine horns using 1-0 Vicho suture.

The robot surgery team is recommended to execute a surgical procedure involving the drilling of a hole measuring 3–4 cm to the left and 1–2 cm away from the navel, followed by the insertion of a 12 mm Trocar. The lens ought to be positioned under continuous monitoring, while two 8 mm Trocars are to be positioned on each side of the lens hole, at an approximate angle of 15° from the foot side, and at a distance of 8–10 cm, serving as access points for mechanical arms. It is of utmost importance to ensure that the distance between each puncture hole is greater than 8 cm. A Trocar measuring 10 mm in diameter was precisely placed at the outer one-third section of the line connecting the right anterior superior iliac spine and the umbilical cord. Additionally, a 5 mm Trocar was positioned between the lens hole and the robotic arm. These particular locations were designated as supplementary access points during the surgical procedure, enabling the utilization of suction devices and non-invasive intestinal forceps (Figure 2). The removal of lymph nodes near the abdominal aorta, specifically in the superior region of the inferior mesenteric artery at the renal vascular level, can be achieved by either rotating the operating table or repositioning the robotic arm apparatus towards the patient’s cranial aspect. Throughout the surgical procedure, it is crucial to securely attach different position fixators, such as the restraint belt, pelvic fixator, shoulder brace, and foot support plate, onto the operating table. Furthermore, it is recommended to utilize sponge pads, silicone pads, and cotton pads for the purpose of securing the patient’s major joints. This comprehensive fixation approach guarantees the patient’s complete immobilization on the operating table, thereby facilitating accurate position adjustments, optimizing the surgical field of view, promoting ease of surgical operations, and reducing the likelihood of compression caused by the robotic arm.

### 3.4. Various Surgical Techniques

#### 3.4.1. The Excision of Para-Aortic Lymph Nodes

Initially, to enhance visibility within the surgical domain, the lateral peritoneum is incised along the left paracolon sulcus, thereby creating a division between the mesentery and the anterior lobe of the renal fascia (also known as Gerota fascia). Subsequently, the descending colon and mesentery are repositioned towards the inner and cephalic directions, thereby exposing the interconnected branches associated with the left renal vein and its corresponding left ovarian vein. Following this, a complete separation is performed between the left ovarian blood vessels and the left ureter, with subsequent outward traction applied to the left ureter. The left ovarian vein is then effectively severed through the utilization of electrocoagulation. The lymphatic tissue bundle located on the left side of the abdominal aorta can be dissected and subsequently removed in a manner that involves excising it from the inner to the outer layers. The lymphatic vessel is elevated, and it is common to encounter lumbar blood vessels on the anterior surface of the lumbar spine. In order to preserve these blood vessels to the greatest extent possible, the pertinent lymphatic tracts are horizontally separated from the abdominal aorta and vertically separated from the spine. During the process of separation, it is imperative to exercise caution in order to prevent any harm to the branches of the inferior mesenteric artery junction. While it is possible for damage to occur to the upper and lower abdominal nerve plexus during this stage, it is not associated with any significant complications. Proceed with the separation of the left renal vein, followed by the ligation of the upper end of the lymphatic vessel, culminating in its removal. It is crucial to exercise special care during the ligation procedure to avoid any harm to the connecting branches between the upper end of the lymphatic vessels and the semiazygos vein.

Subsequently, the lateral peritoneum of the paracolic groove of the ascending colon should be incised until reaching the duodenum. The separation of the ascending colon and its mesentery from the renal fascia (Gerota fascia) should be carried out. As the separation proceeds, the identification of the vena cava and its associated branches, including the right ovarian vein, should be established. Once the right ovarian blood vessel is completely detached from the right ureter, cauterization of the ovarian blood vessel should be performed. Subsequently, the ascending colon and its mesentery should be retracted inward and in a cephalic direction, while the right ureter should be pulled outward. The right lymph node is composed of a pathway running alongside the inferior vena cava (IVC) and a pathway extending across the surface of the IVC and along the abdominal aorta. The anterior luminal tract can be distinguished from the inferior gastric plexus on the ventral side of the aorta and subsequently removed from the inner to the outer region. Typically, it is observed that certain small veins in the tail converge on the surface of the ventral inferior vena cava. Particular attention should be given to the identification and ligation of these veins. The lymphatic vessels in the lower back can be separated from the inferior vena cava, while preserving. The lymphatic tissue situated amidst the major blood vessels is meticulously dissected at the mesenteric artery level. The lateral inferior vena cava and the abdominal aorta are displaced in the opposite direction, thereby isolating the lymphatic tissue between the abdominal aorta and the inferior vena cava from the aforementioned blood vessels. Typically, the lumbar blood vessels traverse the anterior aspect of the lumbar spine.

#### 3.4.2. The Excision of Pelvic Lymph Nodes

There are two essential requirements for conducting pelvic lymph node dissection, namely the separation of the rectal lateral space and the separation of the bladder lateral space. Once the ureter is identified, it should be gently pulled inward to locate the internal iliac artery. Similarly, the umbilical ligament should be pulled inward to identify the external iliac vein. The process of pelvic lymph node resection commences with the removal of the common iliac lymph nodes, which can be further categorized into three distinct sections: superficial, deep, and medial. The superficial iliac lymph nodes receive lymphatic vessels originating from the superficial lymphatic trunk located along the ventral aspect of the external iliac vessels. The superficial layer of the common iliac lymph nodes can be dissected along the extension of the lymph nodes adjacent to the abdominal aorta. The deep iliac lymph nodes receive lymphatic vessels from the deep lymphatic trunk, which consist of obturator lymph nodes and main ligament lymph nodes. The resection of external iliac lymph nodes along the external iliac artery is feasible, as they can be separated from the external iliac artery and lumbar muscle. The preservation of the reproductive femoral nerve is recommended during separation procedures. The iliac vascular lymph nodes can be effectively separated and excised by dissecting between the external iliac vein and the obturator nerve. The obturator nerve can be readily exposed within this anatomical plane. Following the exposure of the inner aspect of the external iliac vein, the obturator lymph nodes can be separated from the psoas major muscle. Alternatively, intermediate techniques can also be employed to achieve their separation and exposure. When the external iliac vein is laterally displaced, the obturator lymph nodes can be fully detached from the external iliac vein. Prior to separating and incising the distal lymph nodes, it is imperative to identify the obturator nerve. By gently pushing the obturator nerve dorsally and delicately pulling the caudal end of the lymph nodes ventrally, the lymph nodes can be separated from the obturator nerve. The primary ligament lymph nodes are situated inferior to the obturator nerve but can typically be preserved in the majority of cases. Proceed with the resection of the obturator lymph node below the external iliac vein. Obturator veins are frequently observed anteriorly, and their preservation or clamping, as well as cutting or coagulation between clips, depend on the size of the blood vessels. Acquainting oneself with anatomy and optimizing surgical techniques enables the removal of retroperitoneal lymph nodes based on the distribution of lymph node clusters. Adhering to this sequence allows for a precise delineation of the boundaries of each pelvic lymph node group, thereby preventing omissions and redundant procedures.

#### 3.4.3. Lymph Node Resection Performed in Various Specific Circumstances

(1) The excision of metastatic lymph nodes

The typical sites of lymph node metastasis in endometrial cancer include the para-uterine, iliac, obturator nerve, uterine artery, periureteral, and para-abdominal aorta regions. In cases where tumor micrometastasis or lymph node dissection has not yet adhered or become fixed, surgical intervention is not challenging. However, it is crucial to ensure complete resection without cutting through the lymph node capsule to prevent implantation. Conversely, when dealing with metastatic lymph nodes that have firmly adhered, there is often significant risk associated with surgery due to the tight adhesion to surrounding blood vessels, nerves, and ureters. Completing open surgery can frequently pose challenges, resulting in a considerable number of cases being left unresolved. In contrast, traditional laparoscopic surgery offers enhanced visualization and magnification, rendering the deep retroperitoneal lymph nodes, obturator fossa (commonly referred to as the “wolf’s nest” during open surgery), and other anatomical structures more superficial. Consequently, nerves and blood vessels become readily discernible. The likelihood of successfully resecting metastatic fixed lymph nodes is significantly enhanced. However, limitations arise from the two-dimensional field of view, which obscures depth perception, the fixed angle of the ultrasound knife, and suboptimal flexibility. Consequently, when removing tightly adhered and fixed metastatic lymph nodes on blood vessels and nerves, caution is exercised to prevent harm. As a result, some metastatic fixed lymph nodes are frequently left intact on the blood vessels and nerves, with a certain probability of inadvertently incising the lymph node capsule. Traditional laparoscopic surgery is challenging to achieve optimal complete resection. In contrast, robotic surgery offers unparalleled advantages in the removal of adhesive and fixed lymph nodes. By utilizing direct three-dimensional imaging, the high-definition field of vision is amplified by a factor of 10 to 15, surpassing the limitations of human visual perception. Consequently, it enables clear visualization of blood vessels, lymph nodes, arteries, veins, and other tissues. The clarity of anatomical exposure is enhanced by the exceptional flexibility of the monopole electrosurgical knife, which enables visualization of the boundaries between metastatic lymph nodes and peripheral nerves and blood vessels from various angles. The implementation of multi field operations frequently leads to the comprehensive eradication of metastatic adhesions and the secure fixation of lymph nodes. During robotic lymph node removal and fixation surgeries, it is crucial to adhere to the principle of prioritizing ease over difficulty, commencing with regions characterized by lax tissue and progressively addressing more challenging areas after achieving clear dissection. Metastatic lymph nodes commonly exhibit a discernible separation from adjacent blood vessels, indicating a lack of tight integration. In cases where frontal removal proves challenging, an alternative approach involves accessing the region between the external iliac blood vessels and the iliopsoas muscle. This technique allows for the exposure of the internal iliac external vein, identification of the obturator nerve, safeguarding of the blood vessels and nerves of the ureter, and comprehensive removal of the lymph nodes to the greatest extent feasible. In order to prevent the implantation of cancer cells in the pelvic cavity or their dissemination through CO_2_ gas, it is imperative to exercise caution and refrain from damaging the capsule of metastatic lymph nodes. Adhering to the principle of tumor-free conditions, it is advised to promptly transfer the excised lymph nodes into a specimen bag to minimize their prolonged presence within the abdominal cavity. Additionally, complete removal of metastatic lymph nodes situated below the inferior vena cava is also recommended.

**Figure 3:**
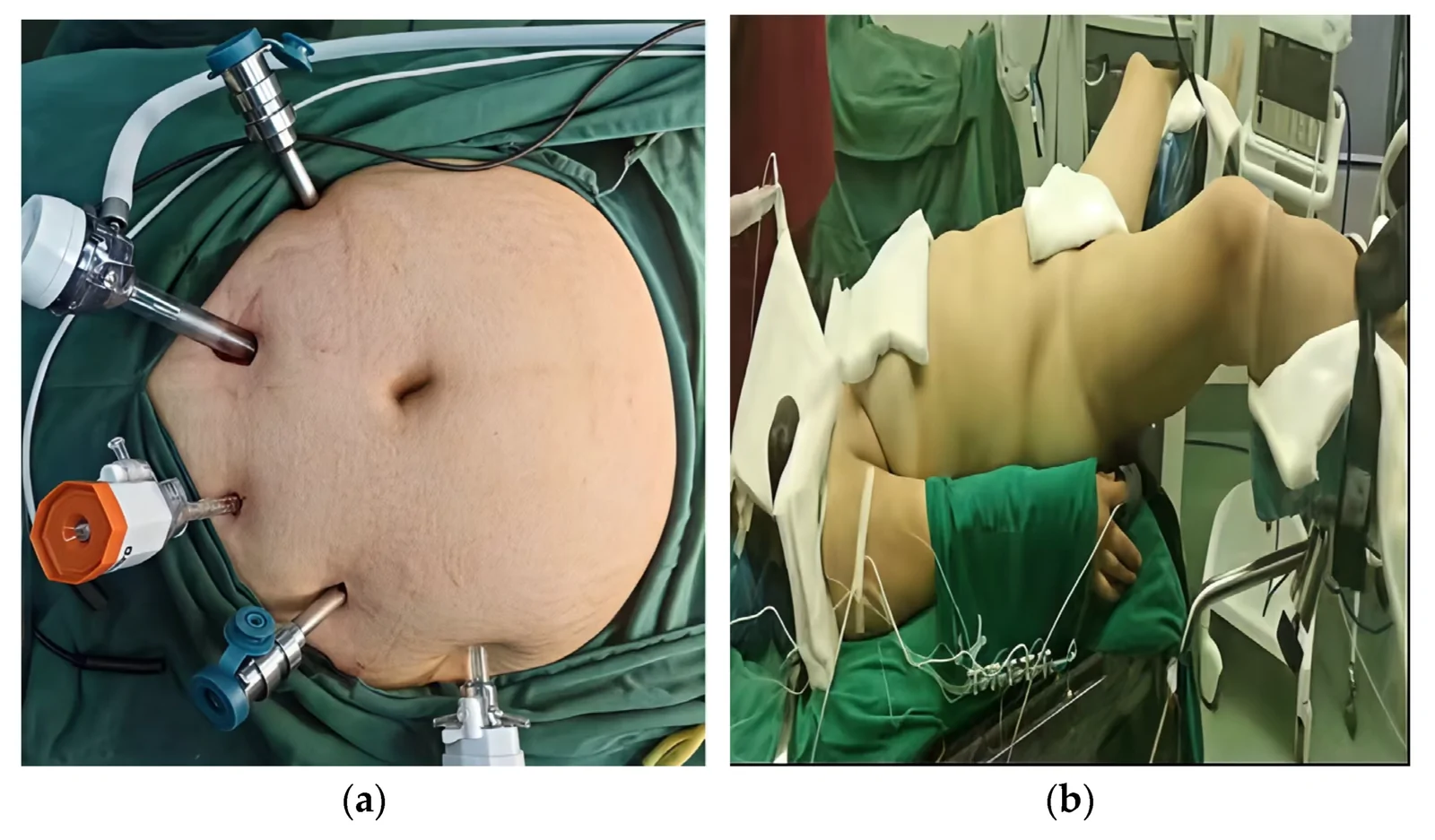
(**a**) Location of puncture hole and (**b**) position and fixation.

In our extensive experience with robotic surgery, we have conducted research on the utilization of a “water cushion” dissection technique. This involves the injection of a water cushion surrounding metastatic fixed lymph nodes to facilitate the separation of lymph nodes. Furthermore, we have submitted an application for the registration of a national utility patent (Figure 3).

(2) The excision of lymph nodes located at the distal portion of the iliac artery

The distal iliac lymph nodes, situated at the outermost region of the pelvic lymph node dissection, pose challenges during their removal due to their distinctive anatomical location. This process is time-consuming and laborious as it can potentially harm nerves and blood vessels. Consequently, the removal of these lymph nodes may lead to a higher occurrence of postoperative lower limb lymphedema and lymphatic cysts in patients. The narrow spaces between blood vessels, nerves, and lymph nodes in this region cannot be accessed or penetrated by conventional instruments or human hands in both laparoscopic and open surgery techniques. However, robot-assisted surgical systems exhibit notable attributes of flexibility and accuracy, enabling them to navigate and operate effectively within confined spaces. Moreover, these systems offer the advantage of providing a comprehensive view of the surgical site, facilitating the identification and removal of smaller and concealed lymph nodes and vessels. Concurrently, the utilization of high-frequency energy instruments is crucial for promptly and precisely clamping residual lymphatic vessels, thereby mitigating the likelihood of postoperative complications such as pelvic lymphatic cysts or lymphatic fistulas.

(3) The resection of the main ligament lymph node

The presence of numerous blood vessels surrounding the primary ligament renders the separation of lymph nodes within the primary ligament susceptible to potential harm to branch veins, thereby compromising the surgical field of vis ion. To ensure an unobstructed surgical field, it is advisable to prioritize the posterior dissection of the main ligament lymph node group. Additionally, the utilization of robotic surgery systems offers distinct advantages, including enhanced magnification, flexibility, and three-dimensional imaging capabilities. The surgeon should possess extensive expertise in the field of robotic surgery and exercise caution during operations, employing delicate movements to prevent any potential damage. In cases involving evident tubular tissue, the utilization of vascular clamps (hemolock clamps) may be considered beforehand, provided that circumstances allow for it. Furthermore, it is imperative to ensure the thorough removal of lymph nodes, particularly in cases where larger lymph nodes cannot be excised entirely. This precautionary measure is essential to prevent the presence of residual lymphoid adipose tissue, as it may contain lymphatic vessels that are challenging to coagulate. Failure to address this issue may result in the gradual leakage of lymphatic fluid post-surgery, thereby increasing the risk of lymphatic fistula or chyle fistula formation.

## 4. Results

There was no statistically significant disparity observed in age, BMI, FIGO staging, pathological type, comorbidities, ECOG score, and history of abdominal surgery between the two groups (*p* > 0.05) (Table 1).

Compared with laparoscopy, robotic-assisted lymphadenectomy was associated with significantly shorter operative time, less intraoperative blood loss, higher total and para-aortic lymph node yields, and a lower overall complication rate. In addition, patients in the robotic group experienced faster recovery, as reflected by earlier anal exhaust, earlier ambulation, shorter postoperative hospital stay, and lower pain scores at 12, 24, and 72 h postoperatively (Table 2). No significant differences were observed in oncologic outcomes (OS and DFS), the need for adjuvant therapy and lymph node pathology positivity between the two groups, although the total cost was substantially higher in the robotic group.

## 5. Discussion

Endometrial cancer is the prevailing gynecological malignancy in developed nations. The absence of a definitive screening program for endometrial cancer in both the general population and specific high-risk groups, coupled with its escalating global incidence rate, poses a significant challenge [16]. Lymph node metastasis serves as the primary mode of metastasis for endometrial cancer [17]. Consequently, surgical intervention necessitates the complete excision of tumor tissue infiltration and the judicious and standardized removal of lymph nodes within the tumor vicinity [18]. The lymphatic metastasis pathway of endometrial cancer is influenced by the tumor’s location, exhibiting a distinct “jumping” pattern of metastasis as opposed to the “stepped” progression commonly observed in cervical cancer. This unique characteristic allows for the bypassing of adjacent pelvic lymph nodes, enabling direct metastasis to the para-aortic lymph nodes located at a distance. Notably, patients diagnosed with endometrial cancer who present with positive pelvic lymph nodes demonstrate a significantly heightened likelihood of also having positive para-aortic lymph nodes [19,20]. In recent years, there has been a growing number of reports on the utilization of sentinel lymph node resection. According to prevailing guidelines, it is advised to employ sentinel lymph node localization resection for lymph node evaluation in cases of low-risk and medium-risk endometrial cancer. Furthermore, it may serve as a viable alternative to lymph node resection for high to medium risk or high-risk clinical stages I-II endometrial cancer [5,21], However, during the study period (2017–2023), both groups of patients were unable to undergo SLN biopsy due to the lack of a fluorescence imaging module in the surgical equipment. In addition, there is a need for prospective studies to generate high-quality evidence regarding the impact of sentinel lymph node localization resection on patient prognosis [22]. The necessity of further retroperitoneal lymph node resection in cases where the sentinel lymph node (SLN) is positive remains unclear [23]. Currently, both the National Cancer Network (NCCN) in the United States and the European Society for Radiology and Oncology (ESTRO) recommend minimally invasive surgery, including robotic procedures, as the preferred treatment for high-risk endometrial cancer, involving systemic lymph node resection [13,24,25,26]. Currently, the International Federation of Obstetrics and Gynecology (FIGO) Gynecological Oncology Staging Committee mandates the inclusion of lymphatic metastasis as a staging criterion for endometrial cancer. Consequently, the management of retroperitoneal lymph nodes must be taken into account when providing treatment to patients with endometrial cancer. Prior to 2006, open surgery was the predominant treatment approach for patients diagnosed with endometrial malignant tumors. While this surgical technique offers enhanced visualization and thorough eradication of the tumor, the substantial incision, particularly in obese and diabetic patients, is associated with a notable rise in complications such as intraoperative bleeding, postoperative pain, incision infection, or incision fat liquefaction [27,28]. In comparison to open surgery, laparoscopic surgery for endometrial cancer yields comparable tumor outcomes, reduced hospitalization duration, decreased blood loss, alleviated pain, and lower perioperative incidence rates [4,29]. Nevertheless, laparoscopic surgery remains constrained by limitations including restricted depth perception, instability in visual field, amplification of tremors, suboptimal ergonomics, an extended learning curve, and vulnerability to fatigue during prolonged surgical procedures. In order to address the limitations faced by conventional laparoscopic techniques, the introduction of robotic surgical systems has been observed. Following Reynolds’ documentation of four instances of robot-assisted staging surgery for endometrial cancer in 2005 [30], there has been a significant surge in clinical research pertaining to robot-assisted endometrial cancer. While the prognosis for advanced endometrial cancer remains unfavorable, patients with early-stage endometrial cancer typically experience more favorable treatment outcomes post-surgery, and may even achieve long-term survival [31]. Enhancing surgical proficiency and establishing uniform surgical approaches hold significant importance. This research delves into the methodologies employed for pelvic and para-aortic lymph node resection in female patients, aiming to identify the most optimal surgical technique with substantial clinical implications. Consequently, this retrospective study incorporates individuals who underwent robot-assisted retroperitoneal lymph node resection for endometrial cancer.

It is important to preface this discussion by acknowledging that our findings derive from a retrospective cohort study with inherent selection bias. Group assignment was influenced by patient preference—a factor that may have systematically allocated healthier, more motivated, or economically advantaged patients to the robotic group, which may be associated with better baseline health and postoperative compliance. This means that the observed perioperative advantages may be partially attributed to patient choice, rather than just technique. The comparative advantages we observed should be interpreted as associations rather than causal effects and the results need to be further validated by prospective studies. In addition, all cases were performed at a single high-volume tertiary center with a dedicated robotic team that had completed structured training. These results may not be generalizable to low-volume centers or surgeons in the early phase of their robotic learning curve.

In our study, it was observed that robotic retroperitoneal lymph node dissection surgery presents several advantages in comparison to laparoscopic surgery. These advantages include a shorter surgical time (165 vs. 180, *p* = 0.03), reduced intraoperative bleeding (45 vs. 65, *p* = 0.04), and a lower incidence of postoperative complications (19 vs. 33, *p* = 0.03). These findings align with the results reported by Giannini A et al. [32]. One possible revision could be: The Da Vinci surgical system offers several significant advantages, primarily attributed to its wider range of motion and improved ergonomics. The utilization of wrist type instruments has notably enhanced the accuracy and flexibility of the system. Notably, the robotic arm’s ability to rotate 540° surpasses that of traditional laparoscopic instruments and even human hands, making it particularly advantageous in patients with combined obesity. This unique feature contributes to the system’s superiority in terms of instrument maneuverability and patient outcomes [33]. The primary distinction between robotic surgery and traditional laparoscopic surgery lies in the absence of direct contact between surgeons and surgical instruments in the former. Conversely, in traditional laparoscopic surgery, the surgeon’s hand directly interacts with the surgical instruments. Consequently, the drawback of traditional laparoscopic surgery is the exacerbation of tremors resulting from unsteady hands [34]. The implementation of a high-definition 3D imaging system enables surgeons to effectively discern tissues and visualize minute blood vessels, nerves, and lymphatic vessels, thereby minimizing inadvertent harm. Additionally, the utilization of robotic surgical instruments facilitates precise and adaptable manipulation, mitigating operational tremors and consequently preventing procedural inaccuracies. In our study, a comparison between laparoscopic surgery and robotic surgery revealed that the latter resulted in a statistically significant increase in the number of lymph node removals (25 vs. 20, *p* = 0.02). This advantage was particularly pronounced in the clearance of lymph nodes located in the superior area of the inferior mesenteric artery, adjacent to the abdominal aorta (5 vs. 3, *p* = 0.04). It is worth noting that the anatomical location of these lymph nodes is relatively deep, extending from the renal artery and vein as the upper boundary to the bifurcation of the abdominal aorta as the lower boundary. Located anteriorly are the duodenum, small intestine, associated blood vessels, and pancreas, while posteriorly lie the vertebral body, lumbar sympathetic trunk and ganglia, ascending lumbar vein, and lumbar muscle. On either side, there exist ureters, ovarian arteries, and kidneys, which present a considerable surgical challenge due to their elevated risk. The comprehensive dissection of para-aortic lymph nodes extends to the midpoint level of the abdominal trunk, encompassing the renal and adrenal blood vessels at the customary high level of para-aortic lymph nodes. The renal blood vessels typically reside at the lower margin of the left renal vein, representing the upper threshold of the lymph node range adjacent to the conventional abdominal aorta. At the level of the inferior mesenteric artery’s root, the traditional low-level para-aortic lymph node level is observed. Several studies have demonstrated that the excision of lymph nodes in proximity to the abdominal aorta at the renal vascular level yields enhanced disease-free survival (DFS) outcomes in comparison to the removal of lymph nodes at the root of the inferior mesenteric artery. Nevertheless, no statistically significant disparity in overall survival (OS) exists between these two approaches [35]. Notably, the utilization of a robot system offers a more distinct visual field and greater operational stability during the clearance of lymph nodes adjacent to the abdominal aorta, as opposed to transabdominal surgery. The wrist turning instrument serves as a valuable tool for identifying and extracting lymph nodes located in confined and inaccessible areas, thereby minimizing the risk of overlooking any nodes. Moreover, robot-assisted laparoscopic surgery offers unparalleled benefits over conventional laparoscopic procedures in terms of excising and securing metastatic lymph nodes. This technique leverages direct view three-dimensional imagery, which enhances the visual field by a factor of 10–15, surpassing the limitations of human vision. Consequently, it enables the precise visualization of anatomical structures such as blood vessels, lymph nodes, arteries, and veins. The anatomical visualization is enhanced, and the unipolar shovel exhibits exceptional flexibility, facilitating the identification of the demarcation between metastatic lymph nodes and adjacent nerves and blood vessels. The utilization of multidirectional and multifield approaches frequently leads to the comprehensive excision of metastatic adhesions and the secure fixation of lymph nodes. The precise manipulation afforded by robotic surgery ensures a high standard of surgical efficacy. There were no statistically significant differences observed in the overall postoperative survival (95.3% vs. 96.8%, *p* = 0.36), disease-free survival (85.3% vs. 87.0%, *p* = 0.58), and postoperative adjuvant chemotherapy (42 vs. 49, *p* = 0.69). However, a notable reduction in surgical complications (19 vs. 33, *p* = 0.03) was observed, indicating an improvement in surgical safety. Furthermore, this study demonstrated that the robotic group exhibited significant advantages in various aspects of postoperative recovery compared to the laparoscopic group. These advantages included shorter postoperative anal exhaust time (47 vs. 55, *p* = 0.02), earlier initiation of bed activity (8 vs. 9, *p* = 0.04), reduced postoperative hospital stay (2 vs. 4, *p* = 0.01), and lower VAS scores (4.0 vs. 4.6, *p* = 0.04, 2.9 vs. 3.4, *p* = 0.03, 1.1 vs. 1.8, *p* = 0.04). These findings indicate that robotic surgery has significant advantages in the perioperative period without sacrificing the safety of oncology in the short to medium term, indirectly emphasize the precision and flexibility of wrist-type instruments in robotic equipment, leading to minimized surgical trauma. However, given the relatively short median follow-up time (38 months median follow-up only reflects early recurrence of cancer), caution should be exercised when evaluating its safety for tumor treatment outcomes, and longer follow-up time (≥5 years) is crucial for confirming tumor non-inferiority.

Despite the advantages of robotic surgery in retroperitoneal lymph node dissection for endometrial cancer, it is important to acknowledge that robot technology is not without imperfections. Presently, the primary limitations of this surgical system include the absence of tactile feedback, which remains a significant technical flaw. Additionally, the substantial physical dimensions of the robot hinder its installation, debugging, disinfection, and maintenance within confined operating rooms. The robot surgical system exhibits a high level of complexity, thereby increasing the likelihood of mechanical failure during its operation in comparison to general endoscopic surgical systems. The system is comprised solely of unipolar, bipolar, and ultrasonic knives, with the latter lacking the capability to bend and offer flexibility in usage. Additionally, it lacks an energy platform for conventional laparoscopy. Furthermore, the surgical procedure necessitates a greater number of puncture incisions than laparoscopy, occasionally requiring the replacement of the trocar site [36,37]. Nevertheless, cost emerges as the primary constraint for robotic surgical systems, primarily attributable to its exclusion from medical insurance coverage. Consequently, the average expenditure incurred by patients for robotic surgery surpasses that of laparoscopic surgery. Nonetheless, our investigation reveals that the substantial decrease in short-term postoperative complications and hospitalization duration within the robotic group contributes to mitigating the long-term expenses associated with robotic surgery. It is anticipated that robotic surgery will experience a similar growth trajectory as traditional laparoscopic surgery in the foreseeable future, characterized by reduced expenses and enhanced cost-effectiveness. Currently, the limited prevalence of robot-assisted surgery in China can be attributed to various factors, including financial constraints, equipment availability, and technological limitations. As the technology of robot surgery continues to advance and surgical instruments undergo further development, it is anticipated that the duration of surgical procedures and average hospitalization periods associated with robot surgery will be reduced. Moreover, a decrease in the occurrence of complications is expected, thereby contributing to the ongoing enhancement of safety and efficacy in the field of robot surgery.

## 6. Conclusions

In brief, robot-assisted retroperitoneal lymph node dissection surgery has emerged as a novel minimally invasive technique in the field of gynecological malignant tumor surgery. Nevertheless, divergent viewpoints exist regarding the optimal selection of surgical approaches, complications, therapeutic efficacy, and postoperative outcomes. Our contention is that robotic-assisted retroperitoneal lymphadenectomy for endometrial cancer is safe, feasible, and offers significant perioperative benefits compared with conventional laparoscopy, including shorter operative time, less blood loss, higher lymph node yield, faster recovery, and a lower complication rate. These advantages are particularly evident in the clearance of high para-aortic lymph nodes and may be further enhanced by standardization of surgical techniques. And adherence to established oncological principles and the involvement of proficient robotic surgeons are imperative in the execution of robotic surgery. By accumulating experience, conducting long-term efficacy observations, and conducting targeted clinical studies with large sample sizes and multicenter randomized controlled trials, individuals can accurately assess the clinical significance of robot-assisted retroperitoneal lymph node resection.

## 7. Patents

This study has registered one patent with the patent number ZL 2019 2 2090596.X, and the patent certificate is shown in Figure 3.

## Figures and Tables

**Table 1:** Demographic characteristics.

	Robot (*n* = 180)	Laparoscope (*n* = 195)	*p* Value
Age (mean, range) (y)	61 (60.4, 42–75)	59 (58.3, 40–69)	0.097
Median BMI (mean, range)	29 (30.5, 24–38)	28 (29.1, 22–36)	0.268
Staging (FIGO)			
Stage IA	123	131	0.925
Stage IB	51	56	0.934
Stage II and above	6	8	0.695
Pathological type			
Endometrioid adenocarcinoma	133	142	0.815
Serous adenocarcinoma	34	37	0.983
Clear cell carcinoma	8	11	0.606
Carcinosarcoma	3	4	0.915
Undifferentiated carcinoma	2	1	0.944
Comorbidity			
Obesity	35 (19.4%)	30 (15.4%)	0.299
Hypertension	95 (52.3%)	96 (49.2%)	0.492
Diabetes	81 (45.0%)	79 (40.5%)	0.380
Other	43 (23.9%)	50 (25.6%)	0.695
History of pelvic surgery	71 (39.4%)	75 (38.5%)	0.845

This table presents a comprehensive overview of the patient characteristics observed in both the robotic and laparoscopic groups. Additionally, the study identifies various comorbidities such as coronary heart disease, cerebral infarction, hyperthyroidism, renal insufficiency, and hypoxemia. Obesity, as defined by a body mass index (BMI) equal to or exceeding 28 kg/m^2^.

**Table 2:** Intraoperative and postoperative conditions.

	Robot (*n* = 180)	Laparoscope (*n* = 195)	*p* Value
Operative time (min)	165 (139–224)	180 (95–190)	0.035 *
Docking time (min)	9 (6–16)	0	0.000 **
Lymph node resection time (min)	105 (82–139)	98 (80–138)	0.015 *
Total number of lymph nodes removed (pieces)	31 (20–43)	22 (17–37)	0.018 *
Number of pelvic lymph node resections (pieces)	25 (18–35)	20 (14–33)	0.020 *
Number of para-aortic lymph node resections (piece)	5 (1–8)	3 (1–6)	0.044 *
Blood loss (mL)	45 (15–70)	65 (15–95)	0.040 *
Anal exhaust time (h)	47 (28–65)	55 (32–75)	0.027 *
First time out of bed activity time (h)	8 (6–19)	9 (6–22)	0.041 *
Postoperative hospitalization time (d)	2 (1–5)	4 (2–9)	0.009 **
VAS			
12 h after surgery	4.0 (2–5)	4.6 (2–6)	0.042 *
24 h after surgery	2.9 (2–4)	3.4 (2–4)	0.038 *
72 h after surgery	1.1 (0–3)	1.8 (0–4)	0.041 *
Complication	19	33	0.032 *
OS (%)	95.3%	96.8%	0.364
DFS (%)	85.3%	87.0%	0.582
Supplemental radiotherapy and chemotherapy	42	49	0.697
suspicious enlarged/fixed lymph nodes	6	7	0.892
Lymph node pathology positivity	8	9	0.932
Total cost (RMB)	63,284 (55,661–83,237)	35,218 (29,812–66,194)	0.001 **

This table intraoperative and postoperative conditions. Values are presented as median (range) or n (%). * *p* < 0.05; ** *p* < 0.01. Robot, robot-assisted laparoscopy; OS, overall survival; DFS, disease-free survival; VAS, visual analogue scale.

## Data Availability

The original contributions presented in this study are included in the article. Further inquiries can be directed to the corresponding author.
